# Current research considering social behavior to improve the welfare of commercially raised dairy calves*

**DOI:** 10.3168/jdsc.2023-0441

**Published:** 2023-11-17

**Authors:** E.K. Miller-Cushon

**Affiliations:** 1 Department of Animal Sciences, University of Florida, Gainesville, FL 32611

## Abstract

Social contact in early life has broad benefits for behavioral development and welfare of the developing dairy calf. The most accessible approach to providing social contact for commercially raised dairy calves is rearing calves in same-age groups, a practice that is growing in popularity. This symposium review highlights developing areas of research relevant for widespread implementation of social housing on commercial farms. I discuss the onset of social behavior in young calves, development of social preferences, implications of calf management and housing for expression of social behavior, individual differences in social behavior, and implications of environmental complexity within the context of social housing. Under both naturalistic and commercial settings, calves interact socially within the first days of life and develop preferences for familiar social companions. Early introduction to social housing appears to benefit the development of social behavior, which may affect integration in later social groups, with potential long-term effects. Housing and management factors affecting socially housed calves have potential to disrupt social synchrony compared with behavior under more natural conditions, which can reduce social lying, cause competition for access to feed, and may have implications for social bond formation and social learning within the social group. Although calves exhibit preferences for familiar individuals and motivation for social synchrony, social behavior is also widely variable between individuals and over time. Individual differences in social behavior may be attributed to personality as well as transient states such as disease or pain, and accommodating individual preferences for social interaction or isolation may be important within groups of larger calves. Throughout this paper, I contrast behavior of commercially raised calves in social housing with behavior of calves under more naturalistic conditions and address both short-term effects for calf development and potential longer-term implications for behavior and welfare. Welfare of commercially raised calves may be improved by refining social housing to better accommodate natural social behavior.

## Graphical Abstract

**Figure:**
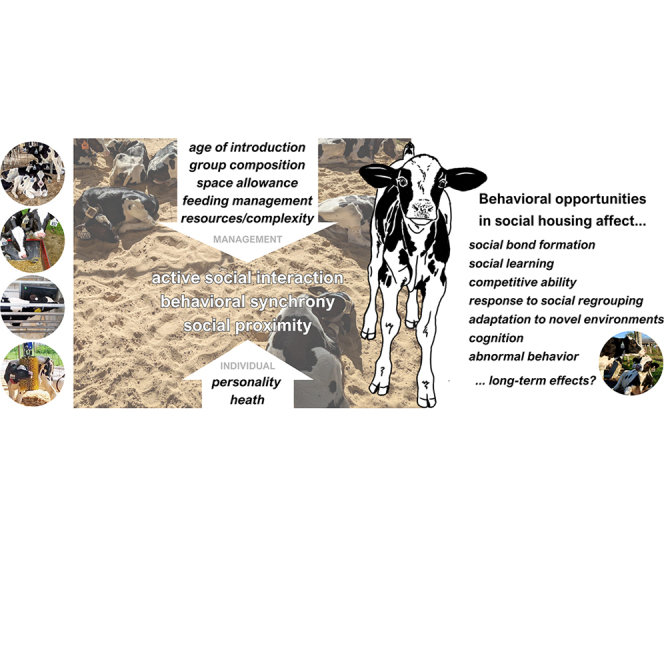

While the dairy industry continues to raise most calves without social contact during the first weeks of life, social housing for young calves is becoming more common. It is well established that housing calves with some type of social contact, compared with individual housing, improves animal welfare. Briefly, in terms of physical health and growth, housing young calves with at least one other calf stimulates solid feed intake and can improve weight gain, particularly during weaning (De Paula Vieira et al., 2010; Miller-Cushon and DeVries, 2016) without compromising health (reviewed by Costa et al., 2016b). There is also evidence to support benefits of social housing for affective state; for example, socially housed calves vocalize less in response to weaning (De Paula Vieira et al., 2010) and may have a more optimistic judgment bias (Bučková et al., 2019). These findings align with evidence across species of detrimental effects of social isolation for broad outcomes including social ability and anxiety (Arakawa, 2018), cognitive development (Kundakovic and Champagne, 2015), and health and longevity (Walker et al., 2012). Although there are varied approaches to rearing calves socially, including with the dam or a foster cow (reviewed by Johnsen et al., 2016), raising dairy calves with one or more similarly aged companions is the most widespread and accessible approach to provide early life social contact within the constraints of commercial dairy production today.

Although social housing unequivocally offers welfare advantages compared with rearing calves in individual pens, there are more nuanced questions of how to best accommodate behavior to improve calf welfare in commercial social housing systems. In this review, I highlight recent research addressing key aspects of dairy calf social behavior and how they relate to environmental and individual factors relevant for commercially raised calves. This review first introduces early social interactions and establishment of social preferences in commercially raised dairy calves. Next, it encompasses current understanding of how common management factors (age of introduction, group composition, and housing environment) interact with the expression of social behavior, including formation of social preferences and behavioral synchrony. It then addresses how social behavior is subject to individual variability, and can relate to individual traits, including personality and health status. Finally, this review discusses broader environmental complexity within the context of social housing, with implications for learning, response to novelty, and development of abnormal behaviors. Throughout, this review considers both immediate effects and potential long-term implications for behavior and welfare. Given the brevity of this symposium paper, this is not a comprehensive literature review of work in this area, but a focused discussion of some of these developing areas of research. Where possible, I highlight recent research related to these topics and identify areas for further research to better understand social behavior and improve the welfare of dairy calves.

While approaches to housing calves socially on commercial farms are definitively different from cattle social groupings under natural conditions, there are some parallels in the expression of social behavior. Under natural conditions, while maternal contact and mother-daughter bonds are critical (Reinhardt and Reinhardt, 1981), calves also interact in same age social groups from a young age. There are reports of large groups resting apart from the dam (Maremma cattle; Vitale et al., 1986) and in small newborn groups under cooperative maternal care (zebu cattle; Orihuela et al., 2021). For example, Maremma cattle spent over 70% of their time away from the dam, socializing with other calves, before 10 d of age (Vitale et al., 1986). Commercially raised dairy calves housed in similar age groups perform social play and allogrooming (reviewed by Costa et al., 2019), key affiliative behaviors that are also observed under naturalistic conditions (Vitale et al., 1986). Social sniffing and licking has been reported at only 2 d of age in pair-housed calves, with calves spending hours of their day engaged in social behavior with their penmate within a few weeks (Duve and Jensen, 2012). Within the first weeks of life, calves are also motivated to access even an unfamiliar calf (Ede et al., 2022). Social contact thus appears important for commercially raised calves from a young age.

Evidence across species suggests that the quantity and quality of social relationships influence individual stress and health status (Cohen, 2004) such that opportunity to form social bonds may be an important predictor of welfare. The formation of social bonds is evident in semi-wild herds of cattle exhibiting persistent preferences for specific social partners when grazing and grooming (Reinhardt and Reinhardt, 1981). Studies of dairy calves separated from the dam at birth also suggest the formation of social preferences between same-age calves that are housed together. Pair-housed calves spent more time near their penmate in a social preference test conducted in a novel arena, with no evidence of social preferences between calves reared in adjacent individual pens (Lindner et al., 2022). Calves housed within the same pen also had greater behavioral synchrony than calves in adjacent individual pens (Duve and Jensen, 2012). Calves raised in pairs prefer to be near their former penmate following introduction to larger groups (Lindner et al., 2021; Zhang et al., 2022). In group-housed calves, the presence of a familiar calf reduces behavioral indicators of fear in an unfamiliar environment (Færevik et al., 2006), supporting the stress-buffering effects of social relationships. While the persistence of social preferences arising from early social interaction has received limited study, Raussi et al. (2010) found that heifers spent the most time near conspecifics they were introduced to in the first weeks of life, and these preferences persisted for over a year, implying the formation of social bonds. Studies of social behavior in adult cows also suggest the formation of social bonds with individuals born around the same time, based on increased social grooming (Sato et al., 1993), closer social proximity (Gutmann et al., 2015), and greater behavioral synchronicity (Gygax et al., 2010).

While social experience under natural settings involves early interaction (Vitale et al., 1986) and opportunities to learn from more experienced social companions (Galef and Laland, 2005), social development in commercially reared calves is subject to age range within the group and timing of grouping. Exposure to an older social model has been found to reduce reactivity to unfamiliar calves (De Paula Vieira et al., 2012b). More experienced social models also benefit social learning, stimulating solid feed intake in young calves (De Paula Vieira et al., 2012a) and influencing foraging behavior in weaned heifers upon introduction to pasture (Costa et al., 2016a; Arrazola et al., 2020). Limited additional work has considered social learning benefits of heterogeneous age groups in commercial social housing systems. Preferential relationships are more likely to occur between similar-age calves in natural settings (Reinhardt and Reinhardt, 1981) and group-housed calves in mixed-age groups also prefer resting next to same-age calves (Færevik et al., 2010), suggesting that contact with more experienced role models may be less related to social bond formation in young calves.

Early social development and opportunity for social bond formation is likely to depend on age of introduction to social housing. For example, Duve and Jensen (2011) describe evidence of preference for a penmate from birth in social preference tests at 5 wk of age, whereas calves paired at 3 wk of age had no preference. Other welfare benefits are associated with earlier introduction to social housing, including increased play and reduced vocalizations throughout the preweaning period (grouped at 3 or 7 d vs. 14 d; Abdelfattah et al., 2018) and reduced vocalizations at weaning (grouped at 5 vs. 28 d; Bolt et al., 2017). Some studies have found that preference for social companions in the first weeks of life may be transitory, but early socialization may provide other social benefits. For example, Lindner et al. (2021) found that calves housed in pairs from birth spent more time lying near their former penmate when initially moved to group pens at 2 wk of age, but preferences did not persist after regrouping 1 wk later. However, following regrouping, previously pair-housed calves had greater total lying time and social rest, even in the absence of a specific preference for their former penmate, suggesting that earlier introduction to social housing may facilitate ability to integrate in novel social groups. More generally, early introduction to social housing may prevent negative effects of early social isolation on the development of social behavior, which are well established in other species (e.g., impaired social recognition in rodents; Arakawa, 2018). While social housing can improve ability to cope with social regrouping compared with individual housing (reviewed by B⊘e and Færevik, 2003), there remain gaps in knowledge of the importance of age of introduction to social housing and potential long-term effects.

Housing factors affecting commercially raised calves may constrain and influence social interactions, including proximity to specific partners and behavioral synchrony, which can elicit competition. Lying synchrony decreases with more restrictive space allowances in group-housed calves (Færevik et al., 2008) and upon movement from pasture to an indoor barn in weaned heifers (Raussi et al., 2010). Synchronous grazing is also evident between preferred social partners under natural conditions (Reinhardt and Reinhardt, 1981), and commercially raised calves also appear motivated to feed socially, which has implications for competition. For example, pairs of milk-fed calves maintain synchronized meals when offered a single teat preventing simultaneous feeding, displacing each other frequently for access to milk, even when milk is supplied ad libitum (Miller-Cushon et al., 2014). Further, weaned calves chose to feed next to a penmate rather than alone, even when feeding spaces were restricted to elicit competition (Miller-Cushon and DeVries, 2016). Competition in group-housed calves fed simultaneously with multiple feeding spaces (e.g., a teat bar or mob feeder) is exacerbated by teat switching, a natural behavior coinciding with reduced milk flow during suckling (de Passillé and Rushen, 2006), which may be reduced by barriers between feeding spaces (Jensen et al., 2008). Feeding synchrony is effectively eliminated through provision of milk autofeeders, which accommodate one calf at a time. However, calves wait to access the feeder, especially when housed in larger groups (Jensen, 2004), suggesting that they remain motivated to feed socially or are experiencing a degree of competition which constraints preferred feeding patterns. Concern has been raised surrounding high autofeeder stocking densities (e.g., 40 calves per feeder; Brscic et al., 2009), which restrict access to the feeder and may limit intake and increase cross-sucking. Mirroring the lack of competition expected in a natural feeding context, best feeding practices for socially housed calves might then accommodate preferences for social feeding while mitigating competition.

Social synchrony and proximity in young ruminants influence opportunities for social learning about feed, such that social contact increases consumption of novel solid feeds (Whalin et al., 2018) and meal frequency of both starter and milk (Miller-Cushon and DeVries, 2016). These beneficial effects are enhanced through access to older animals (e.g., weaned heifers; De Paula Vieira et al., 2012a), although age heterogeneity in groups of preweaning calves may increase competition for milk and negatively affect performance (Færevik et al., 2010). Benefits of early social contact around feeding, including learning about solid feed and improving ability to cope with competition (Duve et al., 2012; Suchon et al., 2023), have potential to mediate longer-term differences in growth of developing dairy heifers. In general, managing calves to accommodate behavioral synchrony allows expression of social preferences and opportunity for social learning, and reduces competition.

While socially housed calves generally exhibit preferences for social synchrony and proximity to familiar animals, social behavior is also subject to individual variability. Evidence across species suggests that social relationships may be influenced by personality (individual behavioral differences which are consistent across time and context), and personality in turn may be shaped by early social experience, particularly in juveniles (Krause et al., 2010). In dairy calves, Lecorps et al. (2019) described individual variability in selectivity of social partners, with an association between social avoidance and pessimism. Consistency of individual differences in social behavior over time has received limited study in dairy calves, although some evidence suggests that centrality in social networks is repeatable across time periods during the preweaning and early postweaning stage (Burke et al., 2024). Adding physical complexity may provide opportunities for individuals to express preferences for social engagement or isolation that mirror more natural environments, including the use of forms of physical separation or a “hide” (reviewed by Spitzer et al., 2022). In group-housed dairy calves, use of a 3-sided physical shelter in the pen was highly variable between individuals, but calves showed individual consistency over a 3-d observation period (Gingerich et al., 2020). Opportunity to express individual preferences for social behavior may be an important welfare consideration for group-housed calves, which warrants further study.

Variability in social behavior between individuals and over time may also be subject to transient internal factors, including health. For example, social withdrawal is commonly described as a component of sickness behavior across species (Dantzer, 2009). In dairy calves, disease has been associated with reduced social behavior, including reduced durations of social grooming and social lying (Hixson et al., 2018), reduced social play (Bertelsen and Jensen, 2019), and reduced centrality in social networks based on frequency of social contacts (Burke et al., 2022) and social proximity (Vázquez-Diosdado et al., 2023). Social connectedness may also relate to disease risk (e.g., *Escherichia coli* colonization in group-housed dairy calves was associated with active social interactions; Tamminen et al., 2020). Individual social behavior may also respond to pain. Following disbudding, group-housed dairy calves increased use of a physical shelter and were more likely to enter it when it was unoccupied (Gingerich et al., 2020), potentially reflecting motivation for isolation or physical protection. Evidence in other species also suggests that the relationship between individual emotional state and social behavior may depend on social context (e.g., primates seek comfort from a familiar companion following an experimental infection; Willette et al., 2007), suggesting that social changes in response to pain or disease may depend on existing social relationships. Larger scale exploration of individual variability in social behavior (e.g., through the use of technology for measuring position and proximity) may lay groundwork for developing novel behavioral indicators of animal welfare.

In addition to accommodating aspects of natural social behavior, social housing for commercially raised calves provides broader opportunities for environmental complexity. Some aspects of environmental complexity are intrinsic to social housing (i.e., social contact and added space), whereas other additional resources may become more feasible or cost effective to provide in conjunction with social housing, such as rotating brushes to stimulate grooming (Horvath and Miller-Cushon, 2019a). Some established benefits of social housing for young calves, including easing adaptation to novel environments, improving cognition, and reducing abnormal behavior, may be enhanced by broader consideration of environmental complexity. First, social contact is known to lessen behavioral and physiological indicators of fear when faced with novelty, across species (reviewed in rodents by Arakawa, 2018) including calves (Jensen et al., 1997). For example, latency to contact a novel object was reduced in group-housed calves that had been housed in pairs prior to grouping rather than individually (Gingerich et al., 2023). However, more extensive studies in rodents suggest that novelty exploration is related to the complexity of the rearing environment, apart from social stimulation specifically (Zimmermann et al., 2001). This is supported in dairy calves by findings that preweaning physical enrichment (stationary brushes, objects for oral manipulation, and scented hay in nets) increased social sniffing and exploration of the environment during postweaning grouping, whereas pair housing alone did not (Zhang et al., 2022).

Cognitive ability may also depend on both early social contact and environmental complexity in general. Cognitive flexibility of dairy calves is improved through social housing (reversal learning ability in a visual go/no-go task; Gaillard et al., 2014; Meagher et al., 2015) as well as potentially other aspects of the calf rearing environment, including dietary complexity (e.g., hay provision; Horvath et al., 2020). There is also evidence of improved object recognition memory associated with both social housing (Gaillard et al., 2014) and physical enrichment in the absence of effects of social contact (Zhang et al., 2022). Long-term effects of early environmental restriction, including social isolation, on cognitive outcomes are established in other species (reviewed by Kundakovic and Champagne, 2015) but remain unreported in dairy cattle.

In addition to influences on cognition and response to novelty, environments which restrict opportunities for motivated behaviors may give rise to abnormal behaviors. Dairy calves are highly motivated to suckle, and insufficient opportunity for natural suckling behavior leads to abnormal oral behaviors including pen-directed sucking, tongue play and rolling, and cross-sucking in socially housed calves. While the performance of these behaviors is closely tied to hunger and suckling opportunities, they are also reduced through behavioral opportunities including those that promote oral manipulation (hay provision; Horvath and Miller-Cushon, 2019b; Downey and Tucker, 2023) and other forms of stimulation, including brush provision (Horvath et al., 2020). Abnormal oral behaviors also occur more often in individual housing, even when accounting for cross-sucking in pair-housed calves (Doyle and Miller-Cushon, 2023). The degree of social isolation may also play a factor (e.g., visual isolation increased pen-directed sucking compared with visual or physical contact; Jensen et al., 1999). Both physical enrichment and pair housing have been found to reduce pen-directed nonnutritive sucking (Zhang et al., 2021) and physical enrichment allowing oral manipulation reduced cross-sucking in group-housed animals (Salter et al., 2021; Zhang et al., 2021), suggesting that environmental complexity of socially housed animals can mitigate development of abnormal oral behaviors. Welfare of commercially raised calves is therefore likely to be improved by considering opportunities for increased behavioral expression in conjunction with social housing.

Calf management decisions may increasingly address behavioral needs of calves, considering widespread concern accommodating “naturalness” in the lives of dairy cattle (Beaver et al., 2020). Research to date suggests that socially housed dairy calves should be managed to accommodate preferred social interactions and behavioral synchrony, which supports the development of social bonds and other benefits of social contact, including social learning. In the absence of providing maternal contact, this may be best achieved by mirroring aspects of natural social behavior: small groups of similarly aged calves, housed from birth in physically complex environments that allow for individual expression of social preferences. While further research linking early life management with long-term behavior and welfare outcomes would be beneficial, social housing and environmental complexity more generally provide cognitive and behavioral benefits in early life with no evidence of detrimental effects. Given the multitude of challenges experienced by a growing dairy heifer, including changes in housing and management, her behavioral and cognitive ability to adapt to novel environments and cope with social stressors may be a strong predictor of welfare which is shaped by early experience.

## References

[bib1] Abdelfattah E.M., Karousa M.M., Lay D.C., Marchant-Forde J.N., Eicher S.D. (2018). Short communication: Effect of age at group housing on behavior, cortisol, health, and leukocyte differential counts of neonatal bull dairy calves. J. Dairy Sci..

[bib2] Arakawa H. (2018). Ethological approach to social isolation effects in behavioral studies of laboratory rodents. Behav. Brain Res..

[bib3] Arrazola A., Dicker K., Vasseur E., Bergeron R. (2020). The effect of early housing and companion experience on the grazing and ruminating behaviour of naïve heifers on pasture. Appl. Anim. Behav. Sci..

[bib4] Beaver A., Proudfoot K.L., von Keyserlingk M.A.G. (2020). Symposium review: Considerations for the future of dairy cattle housing: An animal welfare perspective. J. Dairy Sci..

[bib5] Bertelsen M., Jensen M.B. (2019). Does dairy calves’ motivation for social play behaviour build up over time?. Appl. Anim. Behav. Sci..

[bib6] Bøe K.E., Færevik G. (2003). Grouping and social preferences in calves, heifers and cows. Appl. Anim. Behav. Sci..

[bib7] Bolt S.L., Boyland N.K., Mlynski D.T., James R., Croft D.P. (2017). Pair housing of dairy calves and age at pairing: Effects on weaning stress, health, production and social networks. PLoS One.

[bib8] Brscic M., Gottardo F., Prevedello P., Tessitore E., Cozzi G. (2009). Veal calves’ clinical/health status in large groups fed with automatic feeding devices. Ital. J. Anim. Sci..

[bib9] Bučková K., Špinka M., Hintze S. (2019). Pair housing makes calves more optimistic. Sci. Rep..

[bib10] Burke K.C., do Nascimento-Emond S., Hixson C.L., Miller-Cushon E.K. (2022). Social networks respond to a disease challenge in calves. Sci. Rep..

[bib11] Burke K.C., Gingerich K., Miller-Cushon E.K. (2024). Factors associated with the variation and consistency of social network position in group-housed calves. Appl. Anim. Behav. Sci..

[bib12] Cohen S. (2004). Social relationships and health. Am. Psychol..

[bib13] Costa J.H.C., Cantor M.C., Adderley N.A., Neave H.W. (2019). Key animal welfare issues in commercially raised dairy calves: Social environment, nutrition, and painful procedures. Can. J. Anim. Sci..

[bib14] Costa J.H.C., Costa W.G., Weary D.M., Machado Filho L.C.P., von Keyserlingk M.A.G. (2016). Dairy heifers benefit from the presence of an experienced companion when learning how to graze. J. Dairy Sci..

[bib15] Costa J.H.C., von Keyserlingk M.A.G., Weary D.M. (2016). Invited review: Effects of group housing of dairy calves on behavior, cognition, performance, and health. J. Dairy Sci..

[bib16] Dantzer R. (2009). Cytokine, sickness behavior, and depression. Immunol. Allergy Clin. North Am..

[bib17] de Passillé A.M.B., Rushen J. (2006). Calves’ behaviour during nursing is affected by feeding motivation and milk availability. Appl. Anim. Behav. Sci..

[bib18] De Paula Vieira A., von Keyserlingk M.A.G., Weary D.M. (2012). Presence of an older weaned companion influences feeding behavior and improves performance of dairy calves before and after weaning from milk. J. Dairy Sci..

[bib19] De Paula Vieira A., de Passillé A.M., Weary D.M. (2012). Effects of the early social environment on behavioral responses of dairy calves to novel events. J. Dairy Sci..

[bib20] De Paula Vieira A., von Keyserlingk M.A.G., Weary D.M. (2010). Effects of pair versus single housing on performance and behavior of dairy calves before and after weaning from milk. J. Dairy Sci..

[bib21] Downey B.C., Tucker C.B. (2023). Providing long hay in a novel pipe feeder or a bucket reduces abnormal oral behaviors in milk-fed dairy calves. J. Dairy Sci..

[bib22] Doyle S.B., Miller-Cushon E.K. (2023). Influences of human contact following milk-feeding on nonnutritive oral behavior and rest of individual and pair-housed dairy calves during weaning. JDS Commun..

[bib23] Duve L.R., Jensen M.B. (2011). The level of social contact affects social behaviour in pre-weaned dairy calves. Appl. Anim. Behav. Sci..

[bib24] Duve L.R., Jensen M.B. (2012). Social behavior of young dairy calves housed with limited or full social contact with a peer. J. Dairy Sci..

[bib25] Duve L.R., Weary D.M., Halekoh U., Jensen M.B. (2012). The effects of social contact and milk allowance on responses to handling, play, and social behavior in young dairy calves. J. Dairy Sci..

[bib26] Ede T., Weary D.M., von Keyserlingk M.A.G. (2022). Calves are socially motivated. JDS Commun..

[bib27] Færevik G., Jensen M.B., Bøe K.E. (2006). Dairy calves social preferences and the significance of a companion animal during separation from the group. Appl. Anim. Behav. Sci..

[bib28] Færevik G., Jensen M.B., Bøe K.E. (2010). The effect of group composition and age on social behavior and competition in groups of weaned dairy calves. J. Dairy Sci..

[bib29] Færevik G., Tjentland K., Løvik S., Andersen I.L., Bøe K.E. (2008). Resting pattern and social behaviour of dairy calves housed in pens with different sized lying areas. Appl. Anim. Behav. Sci..

[bib30] Gaillard C., Meagher R.K., von Keyserlingk M.A.G., Weary D.M. (2014). Social housing improves dairy calves’ performance in two cognitive tests. PLoS One.

[bib31] Galef B.G., Laland K.N. (2005). Social learning in animals: Empirical studies and theoretical models. Bioscience.

[bib32] Gingerich K.N., Choulet V., Miller-Cushon E.K. (2020). Disbudding affects use of a shelter provided to group-housed dairy calves. J. Dairy Sci..

[bib33] Gingerich K.N., Lindner E.E., Kalman S., Miller-Cushon E.K. (2023). Social contact from birth influences personality traits of group-housed dairy calves. JDS Commun..

[bib34] Gutmann A.K., Špinka M., Winckler C. (2015). Long-term familiarity creates preferred social partners in dairy cows. Appl. Anim. Behav. Sci..

[bib35] Gygax L., Neisen G., Wechsler B. (2010). Socio-spatial relationships in dairy cows. Ethology.

[bib36] Hixson C.L., Krawczel P.D., Caldwell J.M., Miller-Cushon E.K. (2018). Behavioral changes in group-housed dairy calves infected with *Mannheimia haemolytica*. J. Dairy Sci..

[bib37] Horvath K.C., Allen A.N., Miller-Cushon E.K. (2020). Effects of access to stationary brushes and chopped hay on behavior and performance of individually housed dairy calves. J. Dairy Sci..

[bib38] Horvath K.C., Miller-Cushon E.K. (2019). Characterizing grooming behavior patterns and the influence of brush access on the behavior of group-housed dairy calves. J. Dairy Sci..

[bib39] Horvath K.C., Miller-Cushon E.K. (2019). Evaluating effects of providing hay on behavioral development and performance of group-housed dairy calves. J. Dairy Sci..

[bib40] Jensen M.B. (2004). Computer-controlled milk feeding of dairy calves: The effects of number of calves per feeder and number of milk portions on use of feeder and social behavior. J. Dairy Sci..

[bib41] Jensen M.B., de Passillé A.M., von Keyserlingk M.A.G., Rushen J. (2008). A barrier can reduce competition over teats in pair-housed milk-fed calves. J. Dairy Sci..

[bib42] Jensen M.B., Munksgaard L., Mogensen L., Krohn C.C. (1999). Effects of housing in different social environments on open-field and social responses of female dairy calves. Acta Agric. Scand. A Anim. Sci..

[bib43] Jensen M.B., Vestergaard K.S., Krohn C.C., Munksgaard L. (1997). Effect of single versus group housing and space allowance on responses of calves during open-field tests. Appl. Anim. Behav. Sci..

[bib44] Johnsen J.F., Zipp K.A., Kälber T., de Passillé A.M., Knierim U., Barth K., Mejdell C.M. (2016). Is rearing calves with the dam a feasible option for dairy farms?—Current and future research. Appl. Anim. Behav. Sci..

[bib45] Krause J., James R., Croft D.P. (2010). Personality in the context of social networks. Philos. Trans. R. Soc. Lond. B Biol. Sci..

[bib46] Kundakovic M., Champagne F.A. (2015). Early-life experience, epigenetics, and the developing brain. Neuropsychopharmacology.

[bib47] Lecorps B., Kappel S., Weary D.M., von Keyserlingk M.A.G. (2019). Social proximity in dairy calves is affected by differences in pessimism. PLoS One.

[bib48] Lindner E.E., Gingerich K.N., Burke K.C., Doyle S.B., Miller-Cushon E.K. (2022). Effects of social housing on dairy calf social bonding. Animals (Basel).

[bib49] Lindner E.E., Gingerich K.N., Miller-Cushon E.K. (2021). Effects of early social contact on dairy calf response to initial social grouping and regrouping. J. Dairy Sci..

[bib50] Meagher R.K., Daros R.R., Costa J.H.C., von Keyserlingk M.A.G., Hötzel M.J., Weary D.M. (2015). Effects of degree and timing of social housing on reversal learning and response to novel objects in dairy calves. PLoS One.

[bib51] Miller-Cushon E.K., Bergeron R., Leslie K.E., Mason G.J., DeVries T.J. (2014). Competition during the milk-feeding stage influences the development of feeding behavior of pair-housed dairy calves. J. Dairy Sci..

[bib52] Miller-Cushon E.K., DeVries T.J. (2016). Effect of social housing on the development of feeding behavior and social feeding preferences of dairy calves. J. Dairy Sci..

[bib53] Orihuela A., Pérez-Torres L.I., Ungerfeld R. (2021). Evidence of cooperative calves’ care and providers’ characteristics in zebu cattle (*Bos indicus*) raised under extensive conditions. Trop. Anim. Health Prod..

[bib54] Raussi S., Niskanen S., Siivonen J., Hänninen L., Hepola H., Jauhiainen L., Veissier I. (2010). The formation of preferential relationships at early age in cattle. Behav. Processes.

[bib55] Reinhardt V., Reinhardt A. (1981). Cohesive relationships in a cattle herd (*Bos indicus*). Behaviour.

[bib56] Salter R.S., Reuscher K.J., Van Os J.M.C. (2021). Milk- and starter-feeding strategies to reduce cross sucking in pair-housed calves in outdoor hutches. J. Dairy Sci..

[bib57] Sato S., Tarumizu K., Hatae K. (1993). The influence of social factors on allogrooming in cows. Appl. Anim. Behav. Sci..

[bib58] Spitzer H.B., Meagher R.K., Proudfoot K.L. (2022). The impact of providing hiding spaces to farmed animals: A scoping review. PLoS One.

[bib59] Suchon M., Ede T., Vandresen B., von Keyserlingk M.A.G. (2023). Social housing improves dairy calves’ performance in a competition test. JDS Commun..

[bib60] Tamminen L.-M., Hranac C.R., Dicksved J., Eriksson E., Emanuelson U., Keeling L.J. (2020). Socially engaged calves are more likely to be colonised by VTEC O157:H7 than individuals showing signs of poor welfare. Sci. Rep..

[bib61] Vázquez-Diosdado J.A., Occhiuto F., Carslake C., Kaler J. (2023). Familiarity, age, weaning and health status impact social proximity networks in dairy calves. Sci. Rep..

[bib62] Vitale A.F., Tenucci M., Papini M., Lovari S. (1986). Social behaviour of the calves of semi-wild Maremma cattle, *Bos primigenius taurus*. Appl. Anim. Behav. Sci..

[bib63] Walker M.D., Duggan G., Roulston N., Van Slack A., Mason G. (2012). Negative affective states and their effects on morbidity, mortality and longevity. Anim. Welf..

[bib64] Whalin L., Weary D.M., von Keyserlingk M.A.G. (2018). Short communication: Pair housing dairy calves in modified calf hutches. J. Dairy Sci..

[bib65] Willette A.A., Lubach G.R., Coe C.L. (2007). Environmental context differentially affects behavioral, leukocyte, cortisol, and interleukin-6 responses to low doses of endotoxin in the rhesus monkey. Brain Behav. Immun..

[bib66] Zhang C., Juniper D.T., Meagher R.K. (2021). Effects of physical enrichment items and social housing on calves’ growth, behaviour and response to novelty. Appl. Anim. Behav. Sci..

[bib67] Zhang C., Juniper D.T., Meagher R.K. (2022). Effects of physical enrichment and pair housing before weaning on growth, behaviour and cognitive ability of calves after weaning and regrouping. Appl. Anim. Behav. Sci..

[bib68] Zimmermann A., Stauffacher M., Langhans W., Würbel H. (2001). Enrichment-dependent differences in novelty exploration in rats can be explained by habituation. Behav. Brain Res..

